# Preparing for Virtual Interviews: A Pilot Study to Understand the Needs of Interviewees

**DOI:** 10.7759/cureus.70172

**Published:** 2024-09-25

**Authors:** Jeff Barbee, Richard Thompson, Joanne Vakil, Coranita Burt, Michael Essandoh, Scott Holliday

**Affiliations:** 1 Medical Education and Simulation, The Ohio State University College of Medicine, Columbus, USA; 2 Medical Education and Simulation, The Ohio State University Wexner Medical Center, Columbus, USA; 3 Anesthesiology, The Ohio State University Wexner Medical Center, Columbus, USA; 4 Internal Medicine, The Ohio State University Wexner Medical Center, Columbus, USA

**Keywords:** missing data, recruitment materials, residency interviews, under-represented minorities in medicine (uim), virtual interviews

## Abstract

Virtual interviews play a crucial role in the ranking process. National Resident Matching Program (NRMP) data shows a median number of 23.5 interviews per applicant. Considering this volume of interviews, graduate medical education programs must leverage the virtual pre-interview materials to impact applicants’ attitudes and perceptions of the interview beforehand. This study is a secondary data analysis of an anonymous cross-sectional survey collected during two orientation sessions held in June and July 2022. Responses from 123 residents and fellows rated the usefulness of available print and virtual pre-interview resources. Using a 5-point Likert scale, social media sites scored an average usefulness score of 4.0, while the information packet and program website scored 3.88 and 3.90, respectively. This article provides recommendations for restructuring virtual resources to aid residents and fellows, specifically under-represented minorities in medicine (UiM) applicants, in having a quality interview experience.

## Introduction

The COVID-19 pandemic necessitated a shift towards virtual residency and fellowship interviews. The recent recommendation from the Association of American Medical Colleges (AAMC) states that programs “should share their interviewing plans clearly and early, preferably when application requirements are released” during the 2022 to 2023 residency cycle [1]. This study captured the perceived experiences of residents and fellows with the virtual interview process and the online resources made available to them. Previous research states that medical school applicants found that virtual interviews were not a comparable substitute for a physical visit [2]. Furthermore, some applicants, particularly women and under-represented minorities in medicine (UiM), may be placed at a disadvantage in such an environment [2]. However, the positives and conveniences that virtual interviews provide are notable. The purpose of this study was to (1) determine which resources helped improve an applicant’s virtual interview experiences; (2) identify significant correlations between the different resources; and (3) understand if UiM applicants had significantly different pre-interview and day-of-interview experiences. Additionally, this paper could serve as a model for researchers addressing large portions of missing data.

Background and rationale

Data from the Electronic Residency Application System (ERAS) [3] displayed a trend of rising application submissions from 2019 through 2022. MedPage Today by the AAMC reported an average of nearly 70 applications filed per medical student [4]. The median number of interviews these applicants attended in 2021 was 18 [5] and is now up to 23.5 [6]. Interviews play a crucial role in the ranking process for residency programs. Both program directors (PD) and applicants in Kamboj et al.’s gastroenterology fellowship program survey consider the virtual interview day “a big factor in the final rank list (82% and 56%, respectively)” [7], citing letters of recommendation, academic potential, and interview day performance as the most important factors affecting the program rank list.

With the easement of COVID-19 restrictions, many programs were faced with the question of whether to return to in-person interviews. However, the convenience of virtual interviews is clear: less travel and financial strain, fewer missed days of work, and reduced burden on an individual’s support system [5,7-10]. Interviewees can expect to spend between $1,000 and $5,000 during interview season [11]. Because of these obstacles, virtual interviews have been found to be well-received [7]. Besides reducing numerous burdens, they are reliable and allow applicants to convey themselves from a familiar environment [12,13]. One study also found no advantage for interviewees with an in-person experience compared to those who interviewed using the virtual option [8]. Another study found that virtual interviewees were accepted and matriculated at a higher rate than those who had in-person interviews [14].

For all the benefits that virtual interviews provide, there are several disadvantages embedded in this process besides the common technical difficulties, such as a poor network connection. Applicants often cannot gauge the camaraderie of a team and have noted that making a personal connection may be more challenging in a virtual format [7,8,15,16]. Although other studies have found that virtual interviews provide space for applicants to connect personally, they are also unable to see the facility where they may potentially train and practice or visit the city where they will spend the next several years of their lives [7,9,11,13]. Virtual interviews may remove the ability to personally meet with team members and visit the city, but it has been found that there is less stress in participating in a virtual interview compared to an in-person appointment [11]. Eveland et al. found that virtual interviews allowed the interviewees to be in their homes, where they were comfortable discussing their history and reasoning for wanting to become physicians [14]. These findings seem to contradict the fears of virtual interviews and reassure applicants and interviewers that this format is fair and effective.

The challenges of virtual interviews also extend to faculty. Though this format may be more convenient, it still requires a lot of time [5]. One study found that, on average, each applicant interview requires 5.6 hours of faculty time [10]. In the author's opinion, faculty with diverse backgrounds may be overextended in assembling the necessary representative interview panels, considering the increased number of interviews. Organizing an interview committee that reflects under-represented backgrounds allows applicants to better assess the diversity and culture within an organization. This self-assessment is especially crucial for UiM students when deciding on their rank lists [16]. However, requiring such representation in an interview committee can lead to a more significant burden for diverse faculty members with a “minority tax” [16].

## Materials and methods

This study implements a one-time cross-sectional survey design. Our inclusion criterion included those beginning a residency or fellowship program and was mandated to attend these orientation sessions. Participants were recruited during the opening presentation at the orientation and provided links and quick response (QR) codes to the instrument. They were given approximately 10 minutes to complete the survey. This study collected 135 responses. The processes for handling missing data are outlined below.

Instruments

The residency match survey (RMS) is a modification of a match survey distributed previously (2008-2016) at the same graduate medical institution. The survey consists of 31 items eliciting drop-down, Likert scale, and open textbox responses. A committee of experts used two rounds to review and revise this instrument. These experts included two graduate medical education (GME) administrators and three members from an office that supports medical education research. Items referring to traditional in-person interviews were modified to reflect virtual interviews. The final survey can be viewed in Appendix A. Considering the transition to virtual interviews and the availability of online materials, additional questions on social media and online activities and services were added. The survey constructs focused on the usefulness of pre-interview resources, applicants’ interactions with the institution, and applicant characteristics. The instrument also collected the attitudes of respondents toward the institution’s reputation and the program’s curriculum. The outcome of interest for this study is the item: “Overall, how satisfied or dissatisfied were you with your interview day?” This item was administered using a 5-point Likert scale ranging from 'very dissatisfied' to 'very satisfied' and was recorded to reflect a score of 1 to 5, with 1 representing the 'very dissatisfied' option.

Information packet content

The RMS contained six items for the stem: “Please indicate the usefulness of each component of the information package.” The information packet contained descriptions of benefits, the medical center, the metropolitan area, employment opportunities for spouses, a sample contract, and an outline of appointment criteria. These items used a 6-point Likert scale, and five of them ranged from 'very useful' to 'not at all useful.' The sixth option was 'not applicable/do not recall.' The usefulness items were recoded to reflect a 1-to-5-point scale, while the last option was recoded as missing. These six items were averaged together to create an information packet composite score. We also included items that collected the usefulness of a program’s website, social media, and virtual tour. Each item was structured identically to the information items and was scored in the same manner. 

Missing data

The survey collected 135 responses, of which most contained at least some missing data. Multiple imputations were used due to the potential bias and inaccuracies found using the listwise deletion method, which removes all incomplete observations and uses only those with all necessary data [17]. To help improve the accuracy of missing data, imputation elicits the help of auxiliary variables. These variables are not included in the analysis (i.e., regression models) but provide vital information to estimate the missing data. To help identify potential auxiliary variables, we first removed items that were missing 90% or more observations, which accounted for 10 variables in the dataset. The next step in our process was understanding the completeness of our responses. Twenty-two responses were missing 100% of the data and were removed. After evaluating the data, we removed observations that were missing 86% or more data, resulting in 53 observations being removed. To complete this multiple imputation process, we used the multivariate imputation by chained equations (MICE) package [18].

We began our analyses by conducting pairwise correlations between the variables. We did this to understand if there were any relationships between the resources. Examining these correlations demonstrated applicants' gravitation toward specific resources and their relationship to the interview experience. Next, we used a multiple regression analysis to understand if any of these resources were a reliable predictor for the outcome variable, the day-of experience. We used the imputed datasets to regress the independent variables of the information packet composite score, program's website, social media presence, and virtual tour onto the dependent variable. The final step in our analysis was to compare if UiM students had statistically significantly different experiences compared to their non-UiM peers. The scores we compared were the pre-interview experience and day-of-experience outcomes. 

The final data set contained 82 observations and 16 variables. This included the variables previously covered plus the auxiliary variables of gender and the question, “Did you receive an information packet from the institution before your interview?” The imputed dataset was created using 50 imputations, well beyond the recommended five sets [17,18]. The Institutional Review Board at The Ohio State University deemed this study exempt (study #2022E0779). All analyses were conducted using R Studio (Posit, Boston, MA, USA).

## Results

The survey collected race/ethnicity data, which we then collapsed into a dichotomous UiM item. Participants who identified as either White or Asian were grouped as non-UiM. This decision was made to be consistent with the AAMC's definition of those under-represented in medicine [19]. In total, 34 participants reported themselves as being of non-UiM status, while 10 identified as being of UiM status. Gender was also collected as a demographic variable. Of those who responded, 28 participants identified as female, 14 male, one transgender male, and two preferred not to disclose. Again, we collapsed this item into a dichotomous item using the categories of female and non-female.

The average information packet satisfaction score's average was 3.88 (± 0.11), and the website and social media presence had scores of 3.90 (± 0.12) and 4.00 (± 0.11), respectively. The virtual tour had an average of 3.84 (± 0.12). The day-of experience had a mean of 4.67 (± 0.07) and the pre-interview experience had an average of 4.21 (± 0.09). Item averages were computed using a 5-point Likert scale ranging from a score of 1 to 5. These data are displayed in Table 1.

**Table 1:** Satisfaction with resident resources

Item	Mean (SD)
Information packet	3.88 (.11)
Website	3.90 (.12)
Social media presence	4.00 (.11)
Virtual tour	3.84 (.12)
Day-of experience	4.67 (.07)
Pre-interview experience	4.21 (.09)

The virtual tour and social media presence had the strongest correlation (r = 0.72, p < 0.001) while social media presence was also strongly correlated with a program’s website (r = 0.59, p < 0.001) and pre-interview experience (r = 0.64, p < 0.001). The virtual tour was correlated with a program’s website (r = 0.42, p < 0.05). The day-of experience correlated (r = 0.43, p < 0.05) with the information composite score and a weaker correlation with the pre-interview experience (r = 0.26, p < 0.05). The pre-interview experience also correlated with the information packet (r = 0.43, p < 0.05), virtual tour (r = 0.42, p < 0.05), social media presence (r = 0.64, p < 0.001), and the program’s website (r = 0.69, p < 0.001). The full correlation matrix can be found in Table 2.

**Table 2:** Correlation matrix for pre-interview resource PIE: Pre-interview experience, DOE: Day-of experience *Represents a p < 0.05; ***Represents a p < 0.001

Item	1	2	3	4	5	6
Information packet (1)	-	-	-	-	-	-
Program website (2)	0.213	-	-	-	-	-
Program social media (3)	0.303	0.591^***^	-	-	-	-
Program virtual tour (4)	0.199	0.420^*^	0.724^***^	-	-	-
PIE (5)	0.433^*^	0.687^***^	0.641^***^	0.418^*^	-	-
DOE (6)	0.431^*^	0.236	0.077	-0.259	0.261^*^	-

When regressing the dependent variables on the day-of experience outcome, the information packet and social media presence had the largest, positive coefficients of b = 0.17 and b = 0.18, respectively. This is interpreted as for every point increase in satisfaction toward the resource, we can expect the day-of experience to increase by 0.17 and 0.18 of a point, respectively. The program’s website had a coefficient of b = 0.06, and surprisingly, the virtual tour had a negative coefficient of b = -0.22. None of the coefficients were found to be statistically significant in this model.

The pre-interview experience by UiM status

The UiM pre-interview experience mean was 4.22 (± 0.21) compared to the non-UiM score of 4.24 (± 0.14). Due to the small sample size, we implemented the non-parametric Wilcox signed rank test [20]. Using the imputed data and pooling the results, the average p-value and W values were 0.38 and 658.73, respectively. The range for the W score was as low as 394.5 to the largest value of 932. The p-value ranged from 0.007 to 0.99. After reviewing all 50 iterations, four of these would have been statistically significant (p < 0.05). The effect size ranged from, d = 0.01, 95% CI (0.00, 0.54) to d = 1.00, 95% CI (0.41, 1.57).

The day-of experience by UiM status

The UiM day-of experience average was 4.20 (± 0.33), compared to the non-UiM score of 4.70 (± 0.09). Using the imputed data and pooling the results, the average p-value and W values were 0.20 and 727.3, respectively. The range for the W score was as low as 512.5 to the largest value of 882. The p-value ranged from 0.001 to 0.96. After reviewing all 50 iterations, 20 of these would have been statistically significant (p < 0.05). The effect size ranged from d = 0.04, 95% CI (0.00, 0.49) to d = 0.97, 95% CI (0.43, 1.50). Even though the outcome of this analysis is mixed, this may suggest that UiM status plays a role in the type of resources an applicant seeks. See Table 3 for a comparison of resources by UiM status.

**Table 3:** Mean (standard error) by resource and UiM status UiM: Under-represented minorities in medicine

Classification	Information packet	Website	Social media	Virtual tour
Non-UiM	3.41 (0.20)	3.72 (0.19)	4.07 (0.16)	3.73 (0.17)
UiM	4.70 (0.13)	4.00 (0.20)	3.33 (0.18)	3.00 (N/A)

## Discussion

Participants viewed the information packet, social media accounts, and their program's website favorably. The day-of experience was most positively influenced by the information packet and social media presence. The use of social media can provide insight into the working environment of a residency program. In previous studies, this was a factor for a large majority of applicants as they made their decision [21]. This aligns with previous studies, as residents state that geographic factors help influence their decision [21]. This information packet contained information about the metropolitan area where this institution was located. This may have been especially vital if an applicant did not personally visit the location or city. Notably, the pre-interview experience was also significantly correlated with the day-of experience, albeit moderately. This provides evidence that programs should work to compile a comprehensive packet for applicants to help provide information about the specialty and its surrounding area prior to their interview. The statistically significant correlations between these resources and the pre-interview experience provide evidence that applicants follow these accounts or reference them for desired information.

The virtual tour was found to be significantly correlated with those who utilized the program’s websites and social media accounts. This option may not be desired by all applicants, but those who rely mostly upon social media to collect their information may find great value in this. This somewhat aligns with the findings of previous studies that applicants may not find virtual tours all that helpful [2,15]. Applicants of UiM status rated the virtual tour considerably lower than non-UiM students. This seems to align with past research and the concerns that the virtual process would not serve their needs [2]. Our study found no meaningful correlation between the day-of experience and the virtual tour. The interpretation is that the virtual tours did not provide an accurate or meaningful reflection of the specialty to the applicant. If virtual tours continue, programs will be well served to find ways to engage and showcase the team dynamic with a potential interviewee.

When comparing UiM applicants to non-UiM applicants and their day-of experience and pre-interview experience, we did not find sustained meaningful differences. This contrasts with previous studies, particularly of UiM applicants looking for specific details in a program such as 'fit' [2]. Though this difference was not sustained, programs would be wise to consider the needs of UiM students and ensure available resources that can answer questions that this population of students may have. 

Limitations

Though the survey was presented to attendees early in the morning of the orientation, survey fatigue may have triggered some of the attendees to not complete the questionnaire. Allowing only a narrow window for data collection and not employing standard survey techniques of reminder emails contributed to the low response rate. The respondents for this study are from a convenience sample of a single institution, making it difficult to generalize the findings to the larger population. Threats to internal validity were minimized by using imputed data techniques to estimate missing data. Study bias was minimized by referring only to de-identified data.

This study uses a single time point and therefore does not allow us to measure attitudes over time. Participants likely viewed and utilized the resources of other programs, which could have changed their perceptions of how useful the materials available at this institution were. We also cannot differentiate between specialties. Other potential threats are that this data was self-reported, and the respondents could be influenced by social-desirability bias toward the institution.

## Conclusions

There is a weak, but significant, correlation between the pre-interview experience and the day-of experience. This finding should encourage specialty programs to ensure that all pre-interview resources are current and easy to navigate. Though it may be difficult to dedicate time and resources to each pre-interview resource, our study found clear evidence that the website and an active social media presence may help prepare the applicant to have a positive interview experience.

Future research should focus on evaluating policies regarding restructuring information packets, virtual tours, social media sites, and other pre-interview platforms. Exploring the needs of UiM applicants, including their perceptions of how a welcoming, diverse, and representative community should also be pursued by future research.

## Appendices

Appendix A 

Resident Match Survey 

The asterisk indicates items used for this study.

1. With which GME residency/fellowship program(s) did you interview at (name of institution)?  

2. If you stated "Other" in the last question, please describe the program. 

3. At which hospital did you match for your residency or fellowship? 

4. *Did you receive an information package from (name of institution) before your interview? 

5. How useful was the (name of institution) information package in meeting your overall needs? 

6. *Please indicate the usefulness of each component of the information package (on a scale of 1 to 5 scale or NA): Benefits description; institution description; location/area description; employment opportunities for spouses; sample copy of (name of institution) resident contract; requirements for (name of institution) appointment 

7. Please specify in what ways the information package was useful and not useful. 

8. *Overall, how useful was the residency website (on a scale of 1 to 5 or NA)? 

9. Please specify why you think the residency website was useful or not useful. 

10. *Overall, how useful was the residency program's social media site (on a scale of 1 to 5 or NA)? 

11. Please specify why you think the residency social media site was useful or not useful. 

12. *Overall, how useful was the residency program's virtual tour (on a scale of 1 to 5 or NA)? 

13. Please specify what was the most useful or the least useful on the (name of institution)/ College of Medicine (GME resident/fellow) website. 

14. Please specify what was the most useful or the least useful on the residency program's social media site.

15. Please specify what was the most useful or the least useful on the residency program virtual tour.

16. Which of the following sources did you rely on the most for information about the institution?  

Printed information package; website; program-specific residency website; program-specific residency social media site; program-specific residency virtual tour; all of them equally.

17. Was it easy to contact (name of institution) staff with questions before your interview? 

18. *Overall, how satisfied or dissatisfied were you with the pre-interview process (5-point scale)? 

19. Please specify why you were satisfied or dissatisfied with the pre-interview process. 

20. Concerning your interactions with (name of institution) interviewers, please indicate if you agree or disagree with each of the following statements (5-point scale): The interviewers were courteous; the questions the interviewers asked were appropriate; the interviewers adequately addressed all of my questions; they spent enough time with me; they were helpful in answering my questions; they provided a clear description of their roles and duties; they indicated they were satisfied with their residency/fellowship program. 

21. *Overall, how satisfied or dissatisfied were you with your interview day with (name of institution) (5-point scale)?

22. Please specify why you were satisfied or dissatisfied with your interview day with (name of institution).

23. Concerning the information you received before or during the day of the interview day, please indicate how helpful the information was (on a scale of 1 to 5 or NA): General information on (name of institution), general information on the location of (name of institution) 

24. Please rate each of the following offerings from (name of institution) (on a scale of 1 to 5; NA; do not recall): Resident benefit package (i.e., maternity leave, malpractice insurance); salary; on-call hours and service requirements; call room accommodations; (name of institution) policies (i.e., sexual harassment, physician impairment); (name of institution) counseling assistance; University resources (i.e., libraries, athletic facilities, daycare) 

25. Please rate the (name of institution) GME residency/fellowship program for which you were interviewed based on characteristics of a program that you would want to match in (5-point scale): Overall program quality; variety of caseload; variety of hospitals in rotation schedule; curriculum of residency program; research opportunities; reputation of residency program; quality of teaching staff; reputation of (name of institution), location of (name of institution)

26. Please specify why you were satisfied or dissatisfied with the (name of institution) GME residency/ fellowship program. 

27. Excluding (name of institution), how many residency programs did you interview with? 

28. Did you graduate from (name of institution) College of Medicine? 

29. How old are you? 

30. *To which gender identity do you most identify? 

31. *To which race/ethnicity do you most identify? 
